# The threshold and spatial effects of PM2.5 pollution on resident health: evidence from China

**DOI:** 10.3389/fpubh.2022.908042

**Published:** 2022-08-18

**Authors:** Yuegang Song, Tong Xu

**Affiliations:** School of Business, Henan Normal University, Xinxiang, China

**Keywords:** PM2.5 pollution, health insurance, spatial Durbin model, threshold effect, China

## Abstract

Health capital investment is an integral aspect of human capital investment, and it is vitally important to improve residents' health by encouraging them to maintain insurance. This paper estimates the potential impact of particulate pollution (PM2.5) on health insurance buyers at the city level. Using PM2.5 as a representative air pollution indicator, we construct a threshold panel model and a spatial econometric model based on 2000–2019 panel data from 256 Chinese cities and the health production function to examine the impact mechanism through which PM2.5 pollution causes changes in the number of health insurance buyers. The results indicate that higher PM2.5 pollution significantly increases health insurance buyers in China. Considering the threshold effect, per capita GDP has a nonlinear relationship with an increasing marginal effect on the higher number of health insurance buyers. Due to spatial spillover effects, PM2.5 pollution has an additional impact on the number of health insurance buyers, indicating that a lack of awareness of the spatial correlation will result in underestimating the impact of PM2.5 pollution on residents' health. The robustness of adjacency and geographic distance matrices demonstrates that the regression results are robust and reliable. The findings of this study provide a practical reference for health insurers' development and policymakers' pollution control efforts.

## Introduction

Although the rapidly growing economy in China is substantially improving national strength and citizens' quality of life, environmental challenges are accumulating due to extensive ongoing development. These years of accelerated development have generated frequent nationwide air pollution incidents that have placed residents at risk of health hazards, including higher morbidity and mortality rates, and inequal cognitive abilities and health resources, which are pre-existing concerns at all levels of society (1–4). Hazy days featuring particulate pollution (PM2.5) as the main contaminant have been causing serious public health damage. Research has shown that while PM2.5 is less known than other air pollutants, it makes people extremely susceptible to cardiovascular and respiratory diseases while driving up insurance premiums (5, 6). According to Yin et al. (7), 852,000 of the 1.24 million deaths in China attributable to air pollution are due to PM2.5-related atmospheric pollution. In 2019, per capita life expectancy was 77.3 years, yet healthy life expectancy only stood at 68.7 years in China (8). Judging from the statistics, controlling PM2.5 pollution is of real significance for improving residents' health status.

Most developed countries have established complete health insurance systems. Additionally, people in developing countries are also increasingly concerned about national healthcare systems. However, researchers of healthcare systems in developing countries concentrate the majority of attention on the ‘supply side', for example, proposing improvements in the categorisation of the spectrum of disease by making it more scientific and proposing regulation of the misuse of new medical technologies that result in medical service overuse (9). Nevertheless, scholars seldom consider the impact of environmental pollution, particularly PM2.5 pollution, on the number of health insurance buyers from the ‘demand side'.

While being related to climatic factors, air pollution originates from extensive economic development, low energy efficiency, and insufficient environmental governance. While China has made great advances in energy conservation and emissions reduction in recent years, PM2.5 concentration has reached beyond WHO levels overall, with 81% of the population living in areas where PM2.5 concentration standing above WHO levels (7). Recognising emerging environmental problems as a hindrance to China's sustainable development, the Chinese government is actively working to advance energy efficiency solutions by implementing a range of environmental policies. According to Zhang et al. (10), the rapid development and application of information technology for environmental protection has an increasingly remarkable contribution to environmental governance and surveillance. Hao et al. (11) indicate that the internet is enhancing energy saving and emissions reduction efficiency, directly and indirectly, through technological achievements, energy structure changes, human capital, and economic liberalisation. Hao et al. (12) denote that information and communications technology has risen as a substantial mobilising power for driving smart environmental governance in China.

In conclusion, existing research primarily concentrates on the correlation of air pollution with healthcare spending. As an important indicator reflecting the development of the health insurance sector, the number of health insurance buyers can be used as a basis for the government to strategically develop relevant policies for health insurance to improve healthcare services. With a growing standard of living and increasing awareness of the effect of air pollution, a rising number of individuals will choose to purchase insurance. We select the number of health insurance buyers as an explained variable to examine how it is affected by PM2.5 pollution to apprehend the role of health insurance, optimise premium payments, and improve insurance efficiency. How does PM2.5 pollution impact the number of health insurance buyers with the current circumstances of China's economy? Does PM2.5 pollution have any non-linear spillover effect on the number of health insurance buyers? Are there any spatial differences regarding the impact of PM2.5 pollution? Existing literature rarely examines these questions in a comprehensive and systematic manner; therefore, the impact of PM2.5 pollution on the number of health insurance buyers is of practical significance for improving national public health, contributing to a healthier economy and inspiring policymakers to refine the insurance industry regulation.

The paper offers three potential contributions. First, from the research perspective, we construct a macro examination of the health production function (HPF) of PM2.5 pollution affecting the number of health insurance buyers at the level of prefecture cities, referencing existing literature to analyse spatiotemporal distributions and spatial spillover effects. Second, regarding research methods, we employ panel threshold and spatial econometric models to analyse the nonlinear and spatial spillover effects of PM2.5 on the number of health insurance buyers, enriching relevant empirical studies. Third, for data selection, this paper uses panel data from 256 cities in China from 2000 to 2019 as samples. Compared with provincial data, the research conclusions are more authentic and reliable.

The remainder of this paper is organised as follows. Section 2 Literature review provides a discussion of existing literature. Section 3 discusses the model, variables, and data description. Section 4 elaborates on the empirical results and analyses, and Section 5 concludes.

## Literature review

Since the 1970s, the world has been assailed by environmental pollution concurrent with global economic growth, causing researchers to focus on environmental and public health. Existing literature primarily analyses the health impact of air pollution, medical insurance, and the correlations between air pollution and insurance premiums.

The approach of existing studies predominantly focuses on the impact of air pollution on residents' health from medical and economic perspectives. Out of all studies of environmental health risk, the dose–response relationship in environmental and medical science has the vastest extent of research. This study investigates the dependence of residents' health risks on the probability of air pollution exposure, finding that populations with higher exposure may face higher health risks and hazards (13). Fossati et al. (14) and Klepac et al. (15) determine that pregnant women experiencing long-term exposure to a high concentration of PM2.5 may have an increased risk of lower-than-normal neonatal weight and even neonatal death. According to Jedrychowski et al. (16), prenatal exposure of pregnant women to PM2.5 increases the likelihood of respiratory infection and may even cause early-life respiratory tract diseases to newborns. Kim et al. (17) found that children's prolonged exposure to polluted air may increase the likelihood of asthma, hypertension, obesity, and metabolic disturbance. In addition, an analogous investigation by Schraufnagel et al. (18) attributes mental retardation and developmental delays found in children to air pollution. In studying air pollution, Yang and Pan (19) determine that every 10 μg/m^3^ increase in SO2, NO2, and PM10 concentrations drives up the potential mortality rate of cardiovascular and cerebrovascular diseases by 0.4% (95% CI: 0.1%−0.8%), 1.3% (95% CI: 0.2%−2.4%), and 0.4% (95% CI: 0.2%−0.6%), respectively. Js et al. (20) demonstrate heavy metals, atmospheric particulate matter, and similar air pollutants to have a pathophysiological impact on multiple cardiovascular diseases and the incidence of out-of-hospital cardiac arrest. Brook et al. (21) find PM2.5 pollution to be closely correlated to Parkinson's disease, while also increasing the risk of hospitalisation of cases of myocardial infarction, stroke, and heart failure. According to Chen and Chen (22), PM2.5 pollution is also the main cause for rises in healthcare spending, even more so for males, high earners, populations with higher education, those holding insurance, and senior citizens, who exhibit more susceptibility to air pollution.

The earliest approach to public health from the perspective of economics is the HPF initiated by Grossman (23). The economic mechanism of environmental health risk is primarily embodied in the effect of environmental factors on the health depreciation rate (24). Research on the correlations between the environment and economy has long been limited to using the environmental Kuznets curve framework, in which economic growth before a turning point can trigger rising income effects but does not balance out appreciating trends in environmental health risk. As demonstrated by empirical evidence from China, the family income effect of economic growth alleviates, rather than counteracts, the health damage done by worsening environmental pollution (25). Since the substitution effect of residents' health status far exceeds the income effect, environmental pollution can be considered to have detracted from overall public health security (26). Further macroeconomic research indicates that smoke or dust emissions are significantly negatively correlated with total residents' mortality rate (27). Excessive SO_2_ emissions will raise mortality rates from respiratory diseases and lung cancer, leading to higher medical expenditure (28).

Furthermore, some literature examines the health inequalities induced by environmental pollution and the primary perspective inclines towards higher health risk for economically disadvantaged groups as they are more likely to be exposed to environmental pollution (29). It is apparent that environmental pollution is a momentous transmission mechanism for health inequalities, individuals of different socioeconomic ranks vary in environmental risk aversion capability, and differential exposure to environmental pollution will become a new source of both social and health inequalities (30). Such research also implies that stronger environmental regulation is at the core of pollution exposure reduction and environmental health equity (31, 32).

Health insurance is essential to reducing residents' health costs. A large body of literature investigates the impact of health insurance on health (33–35). To explore this impact in depth, researchers conducted two notable experiments: the RAND Health Insurance Experiment (RAND HIE) between 1974 and 1982 and the Oregon Medicaid Experiment in 2008 (36, 37). In a comparative study, Joseph (38) finds that the RAND HIE lowered the mortality rate of high-risk populations by 10%. Finkelstein et al. (37) determine that the participants in the Oregon Medicaid Experiment benefited from lower insurance premiums and enjoyed better health.

Some researchers investigate the impact of China's universal health insurance on the health of the insured. According to He and Nolen (39) and Wagstaff et al. (40), health insurance helps China reduce the overall morbidity rate while increasing the utilisation rate of preventive medicine. Cheng et al. (41) study China's New Cooperative Medical Scheme (NCMS), determining that while the program significantly involves senior citizens in more daily activities, significantly improves cognitive functions, and diminishes health inequalities, NCMS has failed to lower the morbidity rate. Rokicki and Donato (42) find that NCMS buyers are less likely to have abnormal total cholesterol levels than non-buyers. To improve health insurance system efficiency and address the fragmentation problem, China integrated the NCMS and urban residents' health insurance system into the urban and rural residents' health insurance system. Subsequently, Huang and Wu (43) assert that although the new health insurance system improves the hospital care utilisation rate for middle-aged rural and senior citizens and can subsidise hospitalisation expenses, it only has a limited impact on urban residents. Xie et al. (44) investigate provincial-level panel data and the threshold effect, concluding that commercial health insurance can be interdependent and complementary to social health insurance, revealing no substitution relationship between the two. Chen and Lin (45) demonstrate that the quality sensitivity of patients is high enough that providers should deliver a higher quality of care and reduce the follow-up treatments in order to avoid an overprovision of healthcare services. Concisely, commercial health insurance is of great consequence in lowering residents' health costs, promoting the integration of medical care with insurance, and complementing social health insurance.

Chang et al. (46) find air pollution levels and commercial health insurance maintenance to be significantly correlated. Based on the National Nutrition Survey, Chen and Chen (47) quantify the causal effect of air pollution on health insurance from the perspective of avoidance behaviour. Air pollution primarily affects women, children, senior citizens, high earners, and highly educated citizens. According to Chen and Chen (47), an overestimation of the impact of air pollution on health insurance demand may exist if population migration is also not considered. Regarding the direct impact of air pollution on commercial health insurance maintenance and the moderating effect of residents' risk perception, Yuan and Liu (48) conduct theoretical and empirical research on commercial health insurance maintenance, finding a lagged positive impact of air pollution on commercial health insurance maintenance. Wu et al. (49) use provincial-level panel data to study the direct impact of haze on commercial health insurance development and related spillover effects, concluding that as haze pollution has a significantly positive spatial spillover effect, regional commercial health insurance development will be boosted by the worsening haze pollution status of other regions.

The close perusal of the relevant literature demonstrates that while there are comprehensive studies of the impact of environmental pollution on residents' health, minimal literature considers the impact of air pollution on health insurance. Additionally, most of the related panel data represent only the provincial level, and there is an insufficient number of pollutant indicators. Due to data availability, accuracy, and other limitations, present studies of spatial economics rarely examine the city level. Expanding on existing findings, this paper conducts a comprehensive investigation of PM2.5 pollution and health insurance buyers at the city level by creating a threshold panel model (TPM) and an SEM based on 2000–2019 panel data of 256 Chinese cities, using PM2.5 as the indicator of pollution and the macroeconomic HPF to analyse the mechanism of the impact of PM2.5 pollution on public health at the city level and relevant effects.

## Model, variables, and data description

To test the above research hypothesis, this paper first tests the effects of PM2.5 pollution on the number of health insurance buyers using the following basic model referencing Zhang (50). Add other social factors that affect the number of health insurance buyers, expand the impact of PM2.5 on the number of health insurance buyers, control other variables, and establish a multivariate model.


(1) $$\begin{array}{r} y_{it}=\alpha_{0}+\alpha_{1}PM2.5_{it}+\alpha_{2}X_{it}+k_{i}+\lambda_{t}+\varepsilon_{it} \end{array}$$

where *y*_*it*_ denotes the number of health insurance buyers; *PM2.5* denotes *PM2.5* pollution; *X*_*it*_ denotes the relevant controlled variables, primarily including urbanisation level (*urbanrate*), local fiscal expenditure (*fis_exp*), park green area per capita (*greenarea*), the number of hospital beds per 1,000 people (*hos_beds*), the ratio of the population above 65 years of age (*age*), industrial structure (*second_rate*), and regional GDP per capita (*gdp*). The subscript *i* denotes different cities; *t* denotes different years; α_0_denotes intercept terms; κ_*i*_ and λ_*t*_ denote individual fixed effect and time fixed effect, respectively; and ε_*it*_ denotes the random error term of the model.

Second, we expect the impact of PM2.5 pollution on the number of health insurance buyers to be limited by the regional economic development level. When this exceeds a certain threshold, the number of health insurance buyers will significantly increase, so there may be a nonlinear effect of PM2.5 pollution on the health insurance buyers. In this regard, this paper studies the effect of PM2.5 on the number of health insurance buyers by building a panel threshold effects model based on Hansen's model (51). The threshold level of the economic development of actively insured regions is presented as follows:


(2) $$\begin{array}{r} y_{it}=\beta_{0}+\beta_{1}PM2.5_{it}\left(TV_{it}<\gamma \right)+\beta_{2}PM2.5_{it}\left(TV_{it}\geq \gamma \right)+\\ \;\varphi {\text{X}}_{it}+\nu_{i}+\nu_{t}+\varepsilon_{it} \end{array}$$

where [[Inline Image]] is the threshold value. When the threshold variable [[Inline Image]] exceeds threshold value [[Inline Image]], the impact coefficient of PM2.5 on the number of health insurance buyers is β_2_. When the threshold variable [[Inline Image]] is below the threshold value, and the impact coefficient of PM2.5 regarding the number of health insurance buyers isβ_1_. The above equation only considers a single-threshold value. If there are multiple threshold values, the equation can be further extended, and other variables can be employed as above. To analyse the impact of PM2.5 pollution on the number of health insurance buyers in a panel threshold model, we introduce the threshold variable of economic development level (regional GDP per capita), in which, the higher the economic development level of a country or region, the higher the residents' quality of life and the higher its investment in health insurance.

Finally, to verify the spatial spillover effect of PM2.5 pollution on the number of health insurance buyers, we establish the following spatial econometric model (SEM) to introduce a spatial interaction term between PM2.5 concentration and control variables based on Equation 1:


(3) $$\begin{array}{r} y_{it}=\alpha_{0}+\rho Wy_{it}+\phi_{1}WPM2.5_{it}+\varphi_{1}PM2.5_{it}+\phi_{2}WC_{it}+\\ \;\varphi_{2}C_{it}++\mu_{i}+\lambda_{t}+\varepsilon_{it} \end{array}$$

where *y*_*it*_ is the explained variable; α_0_ is a constant term; ρ is the spatial autoregressive coefficient; *W* is the normalised 256 × 256 spatial weight matrix; *W* is the coefficient of the spatial spillover effect; ϕ_1_ and ϕ_2_ are PM 2.5 pollution and the coefficients of the spatial spillover effect of the related control variable, respectively; and other variables are the same as in Equation 2. Model 3 includes the spatial interaction term of the number of health insurance buyers as the explained variable and the spatial lagged term of PM2.5 pollution as the explanatory variable; therefore, Model 3 is a spatial Durbin model (SDM).

This paper uses the econometric statistical software Stata 16 to conduct the empirical analysis, examining 256 prefecture-level cities in China from 2000 to 2019 as a sample to analyse the impact of PM2.5 on residents' health, as some cities were newly established, and a large amount of data are missing.

Considering that the effect of PM2.5 pollution on residents' health may be reflected by the number of health insurance buyers, this variable serves as the explained variable (*num_insur*) to examine the effect of PM2.5 pollution on residents' health. An increase in the number of health insurance buyers can effectively counteract health hazards of air pollution and improve residents' health. Basic urban employee health insurance buyers include those that purchase basic urban employee health insurance and basic urban and rural resident health insurance. Among them, basic health insurance for urban employees follows the regulations of the People's Republic of China which require insurance to be paid by both the employee and the employer, but in actuality, employees may be unwilling to pay or employers do not pay the premium. The provisions of the health insurance system for urban and rural residents follow the principle of voluntariness. The data are from the statistical yearbooks of each city and the *China City Statistical Yearbook*, and the recorded number of health insurance buyers in various cities.

The explanatory variable is the 2000–2019 PM2.5 pollution (*pm25gm*) of the 256 cities. The data are from the Atmospheric Composition Analysis Group at the Dalhousie University and are rasterised to match the vector-mapped mean concentration values of the 256 prefecture-level cities. Archsmith (52) asserts that, unlike other single pollutants, as a composite measure of air quality, PM2.5 pollution has become one of the primary targets for most researchers; therefore, PM2.5 pollution is the core explanatory variable in this paper. Overall, PM2.5 concentrations in 256 cities in China during the sampling period exhibit a decreasing annual trend, indicating that pollution prevention and control have been somewhat effective.

To analyse the impact of PM2.5 on resident health more comprehensively, the control variables detailed in Table 1 are set in this paper based on the relevant literature (53, 54).

**Table 1:** Description of control variable measurements.

**Variable type**	**Variable**	**Measurement**
Control variable	*Second_rate (%)*	Ratio of the secondary industry to regional GDP
	*Urbanrate (%)*	Ratio of the urban population to the total population
	*Age (%)*	Ratio of population above 65 years of age to total population
	*Gdp (hundred million RMB)*	Regional average GDP
	*fis_exp (hundred million RMB)*	Regional fiscal expenditure
	*greenarea (square metre)*	Per capita park green area (total park green area divided by total population)
	*hos_beds (piece)*	Number of hospital beds per 1,000 people

Basic health insurance is one of the social security systems managed by the government. *fis_exp* can reflect the economic strength of a city, which affects the service quality of health insurance and has a small impact on the willingness of residents to buy health insurance. The *greenarea* indicates the cities' greening circumstances and can represent the quality of the urban environment. Green planting can improve the urban environment, reduce residents' disease incidence rate, and affect residents' health expenditure and willingness to buy health insurance. *hos_beds* can reflect the level of cities' medical resources. Cities with rich medical resources will differ from those with poor medical resources and affect residents' willingness to buy health insurance. As the fossil fuel required by secondary industry is a principal factor of PM2.5 generation, this paper uses *second_rate* to reflect industrial structure. In addition to the above, we include several control variables, including urbanisation level, regional per capita GDP, and the proportion of people over 65 years of age.

The data for this study are selected from 256 Chinese cities, with a sample interval of 2000–2019, and are compiled from the *China Statistical Yearbook*, the *China Health Statistical Yearbook*, the *Chinese City Statistical Yearbook*, and the *China Statistic Yearbook of Environment* in previous years, and missing values were generated applying interpolation and averaging. Table 2 presents the results of the descriptive statistics for each variable.

**Table 2:** Descriptive statistics.

	**Variable**	**Observed value**	**Average value**	**Standard deviation**	**Minimum value**	**Maximum value**
Explained variable	*num_insur*	5,160	89.35	161.11	0.68	1922.25
Explanatory variable	*pm25gm*	5,160	43.56	19.00	4.88	152.15
Controlled variable	*urbanrate*	5,159	0.70	0.35	0.08	0.98
	*fis_exp*	5,160	241.82	494.76	0.07	8351.54
	*gdp*	5,160	3.59	3.16	0.11	46.77
	*second_rate*	5,160	49.64	12.19	8.05	92.30
	*greenarea*	5,160	43.69	76.61	0.19	1352.51
	*hos_beds*	5,160	6.21	2.78	0.36	23.13
	*age*	5,160	8.90	5.26	0.13	81.48
Threshold variable	*gdp*	5,160	3.59	3.16	0.11	46.77

The results in Table 2 demonstrate that the mean value of *urbanrate* is 0.7, the minimum value is 0.08, the maximum value is 0.98, and the standard deviation is 0.35, indicating that there is a substantial urbanisation rate among Chinese cities in both temporal and spatial dimensions. The mean values of hospital beds per 1,000 people (*hos_beds*) and park green area per capita (*greenarea*) are 6.21 and 43.69, respectively, and the respective standard deviations are 2.78 and 76.61, indicating that healthcare level and urban environments also vary considerably from city to city. Other control variables also differ to some extent.

## Empirical results and analyses

Considering the endogeneity of the model, it mainly comes from two aspects. The first is reverse causality, which should not exist in this model, because PM2.5 may increase the number of health insurance buyers, and the increase or decrease of health insurance buyers will not affect PM2.5. Second, due to the different specific conditions of each city, there may be endogenous problems caused by missing variables. Existing research on PM2.5 pollution and public health does not usually consider the economic factors involved in environmental health concerns, resulting in a lack of relevance and effectiveness of environmental and health policy. Based on Grossman's HPF, we further introduce economic, social, and environmental factors to explore the impact of PM2.5 pollution on the residents' health insurance purchases at the urban level.

Our empirical analysis is conducted using the econometric statistical software Stata 16. First, we apply the Hausman test (55) to determine whether to use a random- or fixed-effects model. The statistical results indicate that chi2 (9) = (b-B)'[(V_b-V_B)^(^-1)](b-B) = 301.17 and p = 0.000; therefore, the original null hypothesis that individual and explanatory variables are not correlated must be rejected and a fixed-effects model is adopted.

The regression results in Table 3 demonstrate Model 1 estimates without the inclusion of control variables, and Models 2 and 3 are estimates including some and all control variables, respectively. Table 3 reveals that the regression coefficient of PM2.5 pollution regarding the number of health insurance buyers is significantly positive at the 1% level. The regression coefficient of PM2.5 pollution regarding the number of health insurance buyers decreases slightly after the addition of the relevant control variables but remains significantly positive. The regression coefficient of PM2.5 pollution on the number of health insurance buyers in Chinese urban areas has a consistent positive effect, indicating that PM2.5 has a positive effect on the number of health insurance buyers. One possible reason for this is that as the Chinese economy and the number of educated people continue to grow, an increasing number of individuals are becoming aware of the impact of air pollution, particularly PM2.5, on their physical and mental health. China is now actively addressing the adverse effects of PM2.5, such as closing many corporate high polluters, construction of more parks to improve environments, and the addition of new healthcare facilities to improve healthcare quality. Nevertheless, the impact of PM2.5 on the number of health insurance buyers remains significant, indicating that China's current healthcare resources and efforts to improve the environment are insufficient, and PM2.5 pollution continues to affect health insurance. Pollution remains a critical factor affecting the number of health insurance buyers.

**Table 3:** Baseline regression results of the effects of PM2.5 on the number of health insurance buyers.

**Variable**	**Dependent variable: health insurance buyer number (** ***num_insur*** **)**		
	**Model 1**	**Model 2**	**Model 3**
*pm25gm*	0.049***	0.440***	0.247***
	(0.013)	(0.090)	(0.089)
*fis_exp*		0.056	0.045
		(0.042)	(0.036)
*Age*			0.676***
			(0.231)
*hos_beds*			−2.157***
			(0.543)
*Greenarea*			−0.144***
			(0.015)
*second_rate*			0.295**
			(0.118)
*Urbanrate*			29.14***
			(3.439)
*Gdp*			0.007***
			(0.001)
*Constant*	87.22***	56.68***	94.93***
	(4.129)	(4.133)	(7.860)
City-fixed	Yes	Yes	Yes
Time-fixed	Yes	Yes	Yes
*Observations*	5,120	5,120	5,120
*R-squared*	0.098	0.100	0.207
*Number of code*	256	256	256

The values in the parentheses are standard errors. ***, **, and * indicate that the regression results are significant at the 1, 5, and 10% levels, respectively.

The estimates of the control variables demonstrate that industrial structure (*second_rate*) has a significantly positive effect on the number of health insurance buyers. The economic development level (*gdp*) raises the upper limit of the population's healthcare budget for healthcare, and the number of health insurance buyers increases accordingly. The number of beds per 1,000 people (*hos_beds*) reflects the number of medical resources in the city and has a significant negative impact on the number of health insurance buyers. Larger park green area per capita (*greenarea*) indicates cities' higher greening rate and a better environment because green plants can effectively adsorb particulates in the air and release oxygen through photosynthesis, which is highly beneficial to residents' health and has a significant negative relationship with the number of health insurance buyers. In addition, the effect of regional financial expenditure (*fis_exp*) on the number of health insurance buyers, although positive, is not significant, indicating that the effect is not significant.

To further analyse whether the impact of per capita GDP on the number of health insurance buyers has a non-linear effect of the marginal increase, we must first determine whether there is a threshold effect, and before applying the threshold model, we first use the bootstrap sampling method to test the existence of the panel threshold and determine the number of thresholds. According to our previous theoretical analyses, there may be a nonlinear effect between PM2.5 pollution and the number of health insurance buyers. To reduce the error, this paper adopts Hansen's TPM and uses GDP per capita (*gdp*) as the threshold variable to analyse the effect of PM2.5 pollution on the number of health insurance buyers. The next step is to determine the number of threshold variables, and the bootstrap method is employed to derive the *p*-value and the corresponding F-value. The results are presented in Tables 4, 5. Table 4 indicates that the single-threshold value F is 36.45, which is significant at the 10% level, while the double threshold value F is 12.70 and insignificant. We further undertake a double threshold analysis, as presented in Table 5, indicating that the single-threshold effects model should be used for testing; therefore, GDP per capita serves as the threshold variable and a single-threshold model is used to test the effect of PM2.5 pollution on the number of health insurance buyers.

**Table 4:** Threshold effect test (1).

**Test**	***F*-value**	***P*-value**	**Number of BS**	**Critical values at different level of significance**		
				**1%**	**5%**	**10%**
Single-threshold test	36.45	0.0700	500	60.2881	42.3940	32.9209
Double-threshold test	12.70	0.3880	500	73.0486	38.7507	27.3496
Triple-threshold test	26.71	0.0940	500	25.9805	33.1208	32.9209

**Table 5:** Threshold effect test (2).

**Test**	***F*-value**	***P*-value**	**Number of BS**	**Critical values at different level of significance**		
				**1%**	**5%**	**10%**
Single-threshold test	36.45	0.0760	500	57.7505	40.4457	33.8155
Double-threshold test	12.70	0.4160	500	45.5610	32.5000	25.4028

**Figure 1:**
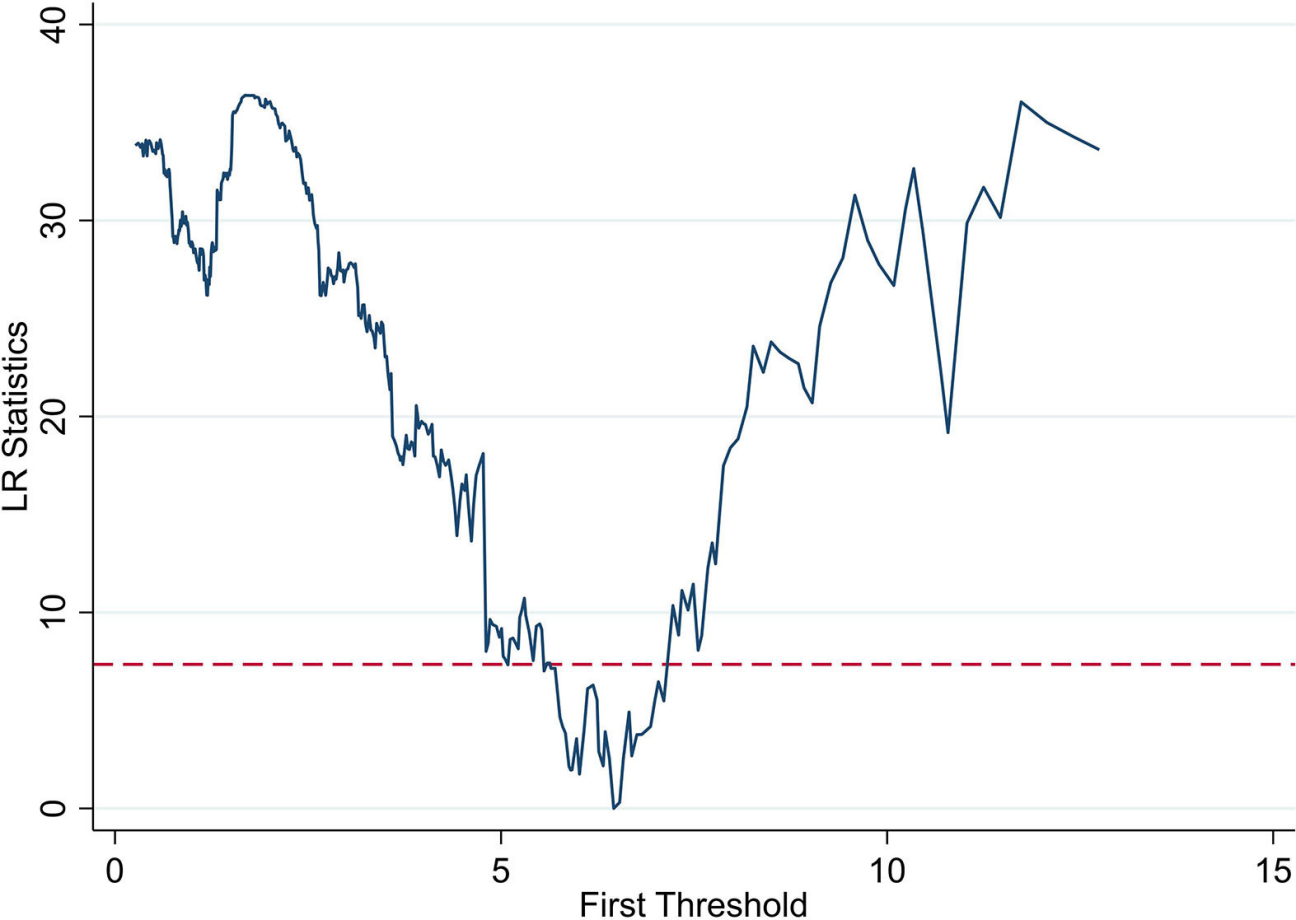
Plot of threshold parameters.

The results of the threshold effect test indicate the presence of a threshold effect on GDP per capita in terms of the number of health insurance buyers, and the results are significant. The likelihood-ratio (LR) plot is shown below (Figure 1), presenting the threshold value and the confidence interval. The dashed line in the graph represents the 95% confidence value which intersects the plot at two points, that is, the confidence interval is 6.0176–6.5357, and the lowest point in the plot is the estimate of the single-threshold value, that is, 6.459.

The effect of PM2.5 on the number of health insurance buyers in China varies depending on the threshold values; therefore, we generate dummy variables based on the threshold values and interaction terms, which regressed. Based on the results of the threshold effect test, we divide GDP per capita into two intervals of d1 (*gdp* < 6.459) and d2 (*gdp* >6.459). The effect of GDP per capita on the number of health insurance buyers is tested based on these intervals. The estimates of the threshold effect in Table 6 indicate that when *gdp* < 6.459, GDP per capita has a significant negative effect on the number of health insurance buyers, indicating that the residents in less developed cities are less willing to buy insurance. The effect of GDP per capita on the number of health insurance buyers is significantly positive when *gdp* >6.459, demonstrating that the buyer number has a non-linear characteristic of marginal increase as per capita income increases and reaches a certain threshold value, which indicates that residents are more concerned about physical and mental health and possess the economic strength to spend on healthcare. As income continues to increase, the positive effect of GDP per capita on the number of insured residents increases.

**Table 6:** Threshold regression results.

**Variable**	**Coef**.	**Std. err**.	***t***	**P>|t |**	**95% conf**.	**Interva1**
*pm25gm*	0.251	0.089	2.83	0.005	0.077	0.425
*xd1*	−1.251	0.508	−2.46	0.014	−2.247	−0.255
*xd2*	0.173	0.062	2.79	0.006	0.172	0.174
*fis_exp*	0.053	0.007	8.12	0.000	0.040	0.066
*age*	−0.684	0.231	−2.96	0.003	−1.137	−0.231
*hos_beds*	−2.183	0.543	−4.02	0.000	−3.248	−1.118
*greenarea*	−0.144	0.015	−9.41	0.000	−0.174	−0.114
*second_rate*	0.280	0.118	2.37	0.018	0.049	0.511
*urbanrate*	32.459	3.645	8.91	0.000	25.314	39.604
*_cons*	94.136	7.860	11.98	0.000	78.727	109.546
*Sigma_u*	125.502					
*Sigma_e*	53.147					
*rho*	0.848					

An SEM analysis is required to test the proposed spatial effect of PM2.5 pollution. First, we conduct a spatial autocorrelation test for the 256 prefecture-level cities in China from 2000 to 2019, and the economic distance weight matrix is imported to calculate the Moran's index (Moran's I) of each city in China from 2000 to 2019. In the univariate spatial correlation test (Table 7), the number of health insurance buyers (*num_insur*) and the Moran's I of PM2.5 pollution are both >0. Although there are some fluctuations, all data are significantly positive at the 1% level, indicating that PM2.5 pollution and the number of health insurance buyers have positive spatial autocorrelation, where the global Moran's I interval of the health insurance buyer number is 0.140–0.180 and the global Moran's I interval of PM2.5 pollution is 0.010–0.055, indicating that the spatial autocorrelation of the number of health insurance buyers is stronger than PM2.5, the aggregation of PM2.5 pollution is poorer, and there is a spatial dependence and spatial heterogeneity between the two (56). If researchers and policymakers ignore the spatial correlations of economic quality development indicators in the empirical analysis, it is likely to lead to a considerable deviation in regression results.

**Table 7:** Univariate global Moran's I.

**Year**	**Buyer (*num_insur*)**	**PM2.5 pollution (*pm25gm*)**
2000	0.140***	0.027***
2001	0.145***	0.018***
2002	0.150***	0.021***
2003	0.155***	0.040***
2004	0.159***	0.019***
2005	0.163***	0.026***
2006	0.169***	0.021***
2007	0.173***	0.022***
2008	0.176***	0.035***
2009	0.179***	0.027***
2010	0.180***	0.010***
2011	0.175***	0.034***
2012	0.176***	0.021***
2013	0.148***	0.035***
2014	0.155***	0.046***
2015	0.158***	0.055***
2016	0.157***	0.048***
2017	0.162***	0.048***
2018	0.169***	0.039***
2019	0.169***	0.051***

***, **, and * mean that the regression results are significant at the 1, 5, and 10% levels, respectively.

**Figure 2:**
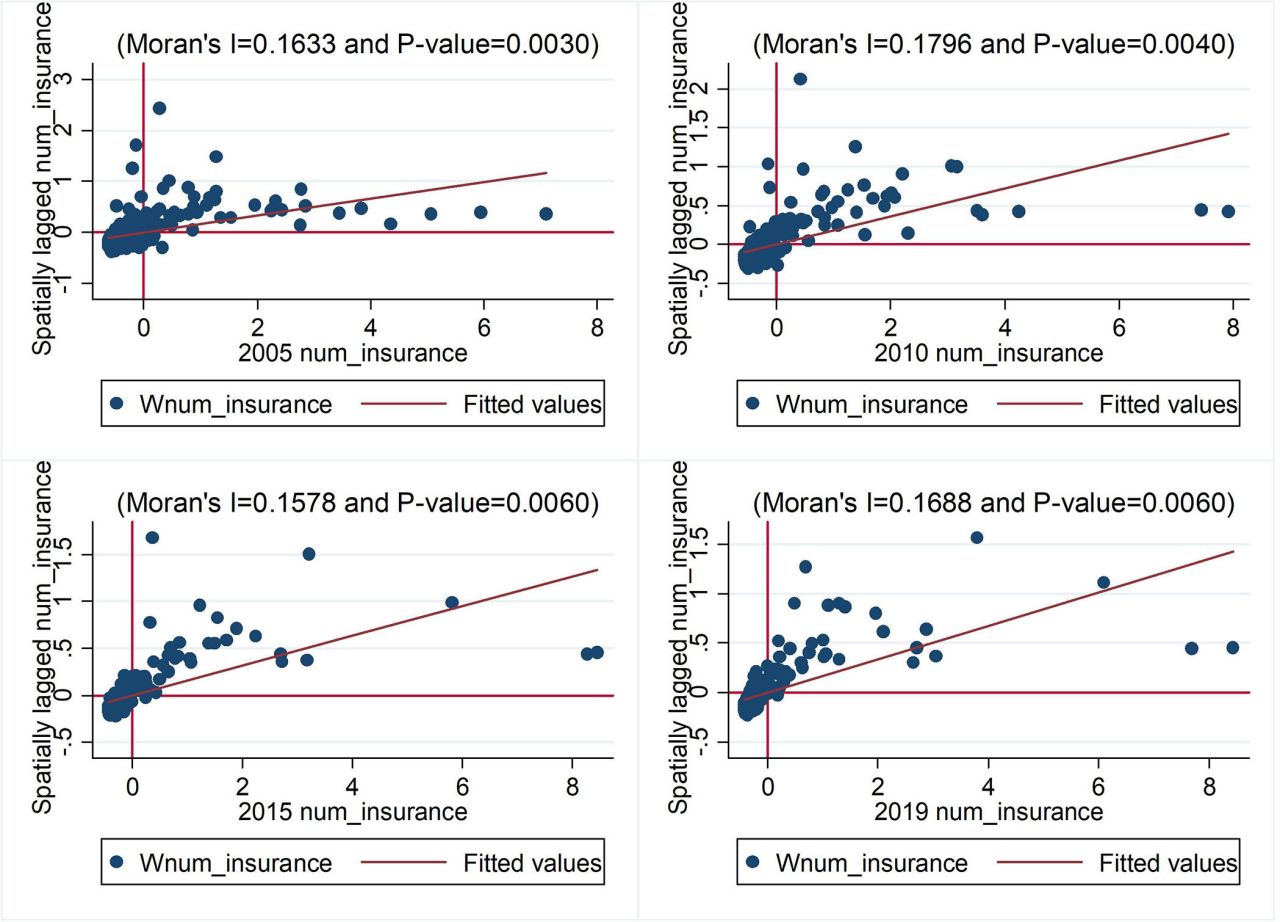
Univariate local Moran's I.

To further examine the aggregation characteristics of the explanatory and explained variables in the local areas, we calculate the local Moran's I of the number of health insurance buyers and PM2.5 pollution. The local aggregation characteristics indicate a positive spatial correlation (Figure 2), and the local autocorrelation of the number of health insurance buyers increases from 0.1633 to 0.1796 from 2005 to 2010, indicating a continuously increasing trend. Notably, the number of buyers is predominantly gathered in the first and second quadrants, primarily in high-high and low-high cities, the number of high-high cities is the largest, and the number of low-low cities is decreasing over time. Analyses of global and local Moran's indices can further reduce the deviation of the spatial econometric regression results.

The results of the global and local Moran's I demonstrate significant spatial correlations; therefore, the application of ordinary panel regression will result in estimated deviations and an SEM should be used. Furthermore, we used LR and Wald tests to ascertain whether the SDM can be degraded to a spatial lag (SAR) or a spatial error model (SEM). The test results are presented in Table 8. The Wald test rejects the original hypothesis at the 1% level, the SDM cannot be degraded to spatial error or spatial lag models, and the results of the LR test are consistent with the Wald test. This indicates that the SDM should be used. In summary, this paper tentatively concludes that it is scientifically appropriate to choose a two-way fixed-effects SDM to estimate the effect of PM2.5 pollution on the number of health insurance buyers.

**Table 8:** Statistics and *p*-values of Wald and LR tests.

**Test method**	**Chic2**	***P*-value**
*Wald-lag*	32.00**	0.0002
*Wald- error*	38.05**	0.0000
*LR-lag*	250.90***	0.0000
*LR-error*	226.22***	0.0000

***, **, and * mean that the regression results are significant at the 1, 5, and 10% levels, respectively.

Given the previous assessment, this paper focuses on the results of the SDM, and Table 9 reveals that the regression results of all three models always show a positive correlation between PM2.5 pollution (*pm25gm*) and the number of health insurance buyers at the 1% significance level. This indicates that PM2.5 pollution leads to an increase in the number of health insurance buyers. The spatial autoregressive coefficient *rho* is positive and significant at the 1% level, indicating a significant regional spillover effect of PM2.5 pollution.

**Table 9:** Regression results of the spatial Durbin model.

**Variable**	**SEM**	**SAR**	**SDM**
*pm25gm*	0.217**	0.236***	0.294***
	(0.093)	(0.085)	(0.008)
*fis_exp*	0.069***	0.064***	0.069***
	(0.006)	(0.006)	(0.007)
*Age*	−0.514**	−0.628***	0.180
	(0.220)	(0.222)	(0.221)
*hos_beds*	−1.933***	−2.103***	−0.344
	(0.524)	(0.522)	(0.524)
*Greenarea*	−0.136***	−0.141***	−0.113***
	(0.015)	(0.015)	(0.015)
*second_rate*	0.277**	0.287**	−0.093
	(0.116)	(0.113)	(0.120)
*Gdp*	0.003**	0.004***	0.006***
	(0.001)	(0.001)	(0.002)
*urbanizationrate*	−40.300***	−26.810***	−15.980**
	(4.101)	(3.310)	(7.427)
*Wpm*25*gm*			−0.372**
			(0.184)
*Wfis*<*uscore*>exp			−0.226***
			(0.025)
*Wage*			0.711
			(0.537)
*Whos*<*uscore*>*beds*			3.900***
			(1.308)
*Wgreenarea*			−0.0216
			(0.070)
*Wsecond*<*uscore*>*rate*			−0.048
			(0.262)
*Wgdp*			0.028***
			(0.005)
*Wurbanrate*			32.130***
			(8.594)
*Lambda*	0.322***		
	(0.029)		
*Rho*		0.278***	0.250***
		(0.028)	(0.028)
*Observations*	5,120	5,120	5,120
City-fixed	Yes	Yes	Yes
Time-fixed	Yes	Yes	Yes
*R-squared*	0.473	0.479	0.586
*Number of id*	256	256	256

The values in the parentheses are standard errors. ***, **, and * indicate that the regression results are significant at the 1, 5, and 10% levels, respectively.

The previous analysis examined the effect of PM2.5 pollution on the number of health insurance buyers under the economic distance matrix, and the results indicate that PM2.5 pollution can significantly increase the number of health insurance buyers. To further verify the robustness of the regression results, adjacency and geographic distance matrices are used. As shown in Table 10, under adjacency and geographic distance matrices, PM2.5 pollution and the spatial autoregressive coefficient (*rho*) mirror previous results, confirming robustness and reliability.

**Table 10:** Regression results of different spatial weights.

**Variable**	**Adjacency matrix**	**Geographic distance matrix**
*pm25gm*	0.088***	0.038**
	(0.033)	(0.015)
*fis_exp*	0.073***	0.076***
	(0.006)	(0.006)
*age*	−0.089	−0.097
	(0.225)	(0.225)
*hos_beds*	−0.990*	−0.995*
	(0.537)	(0.537)
*greenarea*	−0.125***	−0.121***
	(0.0149)	(0.0147)
*second_rate*	−0.0485	−0.135
	(0.123)	(0.124)
*gdp*	0.004**	0.003**
	(0.001)	(0.001)
*urbanrate*	−8.422	−8.116
	(7.719)	(7.526)
*Wpm*25*gm*	0.274*	0.620*
	(0.145)	(0.330)
*Wfis*<*uscore*>exp	−0.370***	0.174*
	(0.136)	(0.099)
*Wage*	−0.537	−2.379
	(2.652)	(2.552)
*Whos*<*uscore*>*beds*	10.18**	−3.225
	(4.477)	(3.649)
*Wgreenarea*	−0.265	0.939***
	(0.682)	(0.327)
*Wsecond*<*uscore*>*rate*	0.336	−1.193**
	(0.812)	(0.596)
*Wgdp*	0.075**	−0.087***
	(0.038)	(0.023)
*Wurbanrate*	9.291	13.060
	(10.180)	(10.610)
*rho*	0.638***	0.695***
	(0.057)	(0.051)
City-fixed	Yes	Yes
Time-fixed	Yes	Yes
*Observations*	5,120	5,120
*R-squared*	0.573	0.558
*Number of id*	256	256

Values in parentheses are standard errors, ***, **, and * indicate the regression results significant at the 1, 5, and 10% levels, respectively.

To further analyse the spillover effect of PM2.5 pollution on the number of health insurance buyers in local and neighbouring regions, this paper estimates the direct, indirect, and total effects of the explanatory variables on the explained variables. The direct effect refers to the spillover effect of the explanatory variable in the local area on the dependent variable, the indirect effect refers to the spillover effect of the explanatory variable in the local area on the explained variable in the neighbouring areas, and the total effect refers to the sum of both. The estimated results are presented in Table 11.

**Table 11:** Direct, indirect, and total effects.

**Variables**	**Health insurance buyers number**
*pm25gm*	Direct	0.018** (0.008)
	Indirect	0.306*** (0.103)
	Total	0.324***(0.112)
*fis_exp*	Direct	0.0543***(0.007)
	Indirect	−0.339***(0.034)
	Total	−0.285***(0.036)
*Age*	Direct	0.115 (0.208)
	Indirect	0.059 (0.630)
	Total	0.174 (0.702)
*hos_beds*	Direct	−0.905* (0.523)
	Indirect	0.250 (1.889)
	Total	−0.655 (2.040)
*greenarea*	Direct	−0.116***(0.014)
	Indirect	−0.202** (0.084)
	Total	−0.318***(0.086)
*second_rate*	Direct	−0.096 (0.121)
	Indirect	−0.054 (0.363)
	Total	−0.150 (0.393)
*gdp*	Direct	0.009***(0.002)
	Indirect	0.052***(0.006)
	Total	0.061***(0.006)
*urbanrate*	Direct	−15.610** (7.754)
	Indirect	50.780** (23.030)
	Total	35.170 (24.800)

The values in the parentheses are standard errors. ***, **, and * indicate that the regression results are significant at the 1, 5, and 10% levels, respectively.

The regression results on the number of health insurance buyers indicate that the direct effect of PM2.5 pollution on the number of health insurance buyers is 0.018, which is significantly positive at the 5% level. Moreover, because of the spatial lag of all explanatory variables, the direct effect also includes the feedback effect of neighbouring areas, indicating the influence of the explanatory variables of neighbouring areas on the number of health insurance buyers in the local area. The feedback effect can be directly obtained based on the differences between the third column in Table 9 (Estimated Coefficients of Each Explanatory Variable) and the direct effects in Table 11; thus, the feedback effect of PM2.5 pollution is 0.016. Similarly, the indirect effect of PM2.5 pollution on the number of health insurance buyers is 0.306, significantly positive at the 1% level. According to this analysis, we can conclude that PM2.5 pollution has a positive spillover effect on the number of health insurance buyers, both inside and among cities.

The significant aim of health insurance is to protect residents' health and prevent tremendous health costs (57). Currently, research on the relationship between PM2.5 pollution and public health focuses mainly on epidemiology and loss of human health and is seldom or never conducted based on economics. We further consider socioeconomic factors to examine how PM2.5 pollution impacts the number of health insurance buyers at the city level.

According to the results, as the core explanatory variable, PM2.5 has a significant impact on the number of health insurance buyers. As the space factor may have spillover effects in terms of the impact of PM2.5 pollution on the number of health insurance buyers, cities should work collaboratively to address atmospheric pollution. Additionally, the secondary industry has a positive impact on the number of health insurance buyers because the consumption of tremendous quantities of fossil energy involves extremely severe pollution; therefore, the higher the proportion of the secondary industry, the greater the environmental stress. Residents will spend more to stay healthy and be more inclined to maintain health insurance. At the same time, rises in residents' income concurrent with rapid economic growth will generate higher inclinations to maintain health insurance.

## Conclusion and policy recommendations

The environment is one of the most critical issues concerning public health, which, in combination with health insurance, is connected to national well-being. There is currently very limited research regarding the influencing factors and levels of health insurance buyers in China, particularly research analysing specific pollution sources such as PM2.5. Based on the data from 2000 to 2019 in 256 Chinese cities, this paper empirically verifies the impact of PM2.5 pollution on the number of health insurance buyers. The relevant results are 3-fold. (1) The estimation of the static panel model demonstrates that the significant environmental pollutant of PM2.5 has significantly increased the number of health insurance buyers. The more serious the air pollution, the higher residents' willingness to buy insurance, indicating a significant positive correlation. (2) The increase of GDP per capita on the number of health insurance buyers has a non-linear relationship with an increasing marginal effect. This paper uses per capita GDP as the threshold variable. When GDP <6.459, per capita GDP has a significant negative impact on the number of health insurance buyers, whereas when GDP > 6.459, the per capita GDP has a significant positive impact on the number of health insurance buyers. (3) The spatial econometric analysis demonstrates that PM2.5 pollution can increase the number of health insurance buyers in the local city with a significant spatial spillover effect, indicating that PM2.5 pollution in a local city has a significant positive effect on the number of health insurance buyers in neighbouring cities.

Based on the empirical analyses above, the paper proposes three countermeasures. First, because environmental pollution contributes significantly to the growth of the number of health insurance buyers, the government should persistently focus on atmospheric pollution control action plans by codifying laws on environmental protection, energy conservation and emissions reduction, regulating corporate production and operational activities, levying pollution tax as soon as practicable, and limiting the excessive growth of insurance premiums. Insurers can introduce green insurance to strengthen environmental pollution risk management, minimise pollution incidents, and safeguard the interests of those affected by environmental pollution. It is essential to make the most of the internet to control pollution, conserve energy, reduce emissions, and optimise resource allocation efficiency.

Second, the government should continue to vigorously develop the economy and enhance public health awareness. The impact of PM2.5 pollution on the number of health insurance buyers has a non-linear relationship with an increasing marginal effect. When the per capita GDP is less than the threshold of ¥64,590, the public will not have a strong willingness to buy health insurance. When the per capita GDP exceeds the threshold value, willingness to buy insurance and the number of buyers will continuously increase. Only continued vigorous economic development, improving incomes, and enhancing public health awareness will increase the number of individuals willing to buy health insurance to ensure public health.

Third, air pollution control requires mutual cooperation and collaborative governance among different cities. The number of health insurance buyers has an autocorrelation with PM2.5 pollution at the city level. According to the global and local Moran's I, the concentration of PM2.5 pollution is poor, while the number of health insurance buyers is relatively strong. The impact of the core explanatory variable, PM2.5 pollution, will have a long and dynamic influence on the number of health insurance buyers. According to the autocorrelation and the lag verification, the influence of PM2.5 pollution has a significant spatial spillover effect on the number of health insurance buyers; therefore, both local city efforts and mutual cooperation and collaborative governance of all neighbouring cities are needed to prevent and control air pollution. Cities with more developed economies can have a leading influence by providing technology and funds to help underdeveloped cities eliminate and upgrade outdated industries to achieve coordinated development in the region.

It is true that there are some deficiencies in predicting the development of the medical insurance industry through PM2.5. When selecting the control variables, only the aging population is considered, and children are also relatively vulnerable to pollution. Due to the unavailability of data, this paper does not use them. In addition, education level and consumption characteristics are also one of the factors affecting the number of health insurance buyers. To this end, we propose future research topics. First, the research adopts a variety of variables for measurement and analysis. For the data that is difficult to obtain from the database, the survey method can be used. Second, to further analyse the impact mechanism of PM2.5 on medical insurance.

## Data availability statement

The original contributions presented in the study are included in the article/supplementary material, further inquiries can be directed to the corresponding author/s.

## Author contributions

YS: conceptualization, methodology, formal analysis, writing—original draft preparation, and funding acquisition. TX: data processing, formal analysis, and writing—original draft preparation. All authors contributed to the article and approved the submitted version.

## Funding

This research was funded by the financial support of the National Social Science Foundation (The impact of heterogeneity of regional trade in services agreements on the reconstruction of global value chain of China's manufacturing industry; Project No: 20BJY091) and Innovative talents in philosophy and social sciences in universities and colleges in Henan Province (Project No: 2022-CXRC-29).

## Conflict of interest

The authors declare that the research was conducted in the absence of any commercial or financial relationships that could be construed as a potential conflict of interest.

## Publisher's note

All claims expressed in this article are solely those of the authors and do not necessarily represent those of their affiliated organizations, or those of the publisher, the editors and the reviewers. Any product that may be evaluated in this article, or claim that may be made by its manufacturer, is not guaranteed or endorsed by the publisher.
